# Quality of Medical Advice Provided Between Members of a Web-Based Message Board for Patients With Implantable Defibrillators: Mixed-Methods Study

**DOI:** 10.2196/11358

**Published:** 2018-12-04

**Authors:** Christopher E Knoepke, D Hogan Slack, M Pilar Ingle, Daniel D Matlock, Lucas N Marzec

**Affiliations:** 1 Division of Cardiology School of Medicine University of Colorado Denver, CO United States; 2 Adult & Child Consortium for Outcomes Research & Delivery Science School of Medicine University of Colorado Denver, CO United States; 3 Data Science to Patient Value Initiative School of Medicine University of Colorado Denver, CO United States; 4 School of Medicine University of Colorado Denver, CO United States; 5 Division of Geriatric Medicine School of Medicine University of Colorado Denver, CO United States; 6 Geriatric Research Education and Clinical Center Veterans Affairs of Eastern Colorado Denver, CO United States

**Keywords:** education of patients, information sharing, implantable cardioverter-defibrillator, data accuracy

## Abstract

**Background:**

Patients use Web-based medical information to understand medical conditions and treatments. A number of efforts have been made to understand the quality of professionally created content; however, none have described the quality of advice being provided between anonymous members of Web-based message boards.

**Objective:**

The objective of this study was to characterize the quality of medical information provided between members of an anonymous internet message board addressing treatment with an implantable cardioverter-defibrillator (ICD).

**Methods:**

We quantitatively analyzed 2 years of discussions using a mixed inductive-deductive framework, first, for instances in which members provided medical advice and, then, for the quality of the advice.

**Results:**

We identified 82 instances of medical advice within 127 discussions. Advice covered 6 topical areas: (1) Device information, (2) Programming, (3) Cardiovascular disease, (4) Lead management, (5) Activity restriction, and (6) Management of other conditions. Across all advice, 50% (41/82) was deemed generally appropriate, 24% (20/82) inappropriate for most patients, 6% (5/82) controversial, and 20% (16/82) without sufficient context. Proportions of quality categories varied between topical areas. We have included representative examples.

**Conclusions:**

The quality of advice shared between anonymous members of a message board regarding ICDs varied considerably according to topical area and the specificity of advice. This report provides a model to describe the quality of the available Web-based patient-generated material.

## Introduction

The vast majority of adults use the internet to research health issues to inform decisions, including whether or not to accept certain tests, medications, or devices[1,2]. Sources of Web-based information include both professionally created websites (eg, WebMD, Mayo Clinic, and industry materials) and user-created content on social media [3]. Web-based medical information fills a critical need for patient education, decision making, and emotional support [4,5]. Therapeutic interventions in many areas of medicine are becoming more complex, and patients forget 40%-80% of the information provided during medical appointments [6]. In contrast, patients can access Web-based information at any time and review it indefinitely. Web-based information fills the educational needs of patients outside of appointments as patients report acting on advice they find [7] and report being satisfied with the information and support they receive through Web [8].

The quality of information that patients encounter on Web varies, including the materials created by professionals. One investigation found that internet resources for ventricular assistance device candidates universally discussed the benefits of therapy, but only half reported risks and only 2 (of 77) mentioned palliative care or hospice[9]. Another examination of webpages of 262 transcatheter aortic valve replacement centers found that all discussed benefits of treatment, but only 26% mentioned any risk [10]. Such limitations within resources created by professionals are likely reflected in information shared between anonymous members of Web-based communities, where patient users logically have less access to the population-based information needed to contextualize advice about complex therapies. Nevertheless, patients who engage in a comprehensive Web-based search will encounter both forms of information.

We chose implantable cardioverter-defibrillators (ICDs) as a model to explore the quality of user-provided information appearing on medical message boards. Decisions regarding whether or not to implant an ICD involve trade-offs. ICDs may lengthen patients’ lives, but have potential risks including infection, lower quality of life, increased hospitalizations, and potential suffering at the end of life [11-13]. Ongoing self-management and decision making are critical in ICD care. Therefore, it is important to ensure the accuracy of advice being acted upon by patients. While our prior work demonstrates that patients report learning about their ICDs from internet message boards [4], the quality of this information is unknown. This project sought to characterize the quality of medical information provided between commenters on an internet message board for patients with ICDs.

## Methods

We utilized a mixed inductive-deductive approach for characterizing and quantifying the quality of medical information shared on an ICD message board. This approach was adapted from one used previously by our group [14]. To focus the content of discussions under analysis and allow for our ability to acquire permission to conduct the described analysis from the site’s webmaster, we limited our search to comment threads appearing on 1 ICD-specific message board. The message board itself (which will not be identified per our agreement with the webmaster) required users to register a username and email address in order to compose posts or answer others’ questions. No member of the research team had known relationships with the commenters, and no attempt was made to identify or contact commenters (whose posts were labeled with self-chosen avatars).

We included all discussions posted between January 1, 2015, and December 31, 2016. Each discussion was uploaded into Dedoose analytic software v 7.1.3 (SocioCultural Research Consultants, Los Angeles, CA) to facilitate team-based analysis. The project was deemed exempt by the local Institutional Review Board.

The analysis was conducted using a progressive deductive, inductive, and quantifying process adapted from our earlier inductive-deductive toolkit [14-15]. After converting all discussions appearing on the message board for analysis, the discussions were coded using a two-stage process, each of which was double coded by at least two members of the authorship team. First, the complete Web-based discussion threads were deductively coded for instances in which one commenter provided another with any form of medical advice. This included coding by two primary analytic team members (CK and HS), with any differences adjudicated by team consensus. Next, the primary author and a board-certified cardiac electrophysiologist (CK and LM, respectively) inductively coded each instance in which medical advice was provided by one commenter to another, creating a framework for analyzing both the topic discussed and the quality of advice. The resulting quality categories included the following: (1) Generally appropriate; (2) Controversial; (3) Inappropriate for most patients; and (4) Without sufficient context to support. Finally, we quantified the proportions of the quality of advice provided between commenters within each topical area.

## Results

### Advice Provided

The total corpus of data included 127 threaded discussions, having been composed by users with 234 unique avatars. During the study period, users posted an average of 2.74 (median 1) comments. Within these discussions, we identified 102 separate instances in which one member provided advice to another. We excluded 20 comments that discussed psychosocial adjustment to ICD placement or shock, leaving 82 pieces of explicit medical advice.

### Topical Areas and Quality

Commenters provided advice in 7 conceptual areas: (1) Device information (n=19); (2) Programming (n=16); (3) Cardiovascular disease (n=9); (4) Procedures (n=6); (5) Lead management (n=4); (6) Activity restriction (n=15); and (7) Management of other conditions (n=13). The overall quality of advice provided was mixed, with 50% (41/82) advice deemed generally appropriate, 24% (20/82) inappropriate for most patients, 6% (5/82) controversial, and 20% (16/82) without sufficient context.

**Table 1:** Quality of information by topical category.

Topic category	Total, N	Generally appropriate, n (%)	Controversial, n (%)	Inappropriate, n (%)	Without sufficient context, n (%)
Device information	19	12 (63)	0 (0)	4 (21)	3 (16)
Programming	6	7 (41)	0 (0)	3 (18)	6 (35)
Cardiovascular disease	9	5 (56)	0 (0)	1 (11)	3 (33)
Lead management	4	2 (50)	2 (50)	0 (0)	0 (0)
Activity restriction	15	10 (67)	0 (0)	5 (33)	0 (0)
Procedures	6	2 (33)	0 (0)	4 (67)	0 (0)
Other disease management	13	3 (23)	3 (23)	3 (23)	4 (31)

However, the proportionate quality of advice provided within each of these categories varied considerably (Table 1).

#### Device Information

Information pertaining to ICD devices themselves, including basic functionality, battery life, and typical care processes, was either generally appropriate (12/19, 63%) or inappropriate (4/19, 21%). This advice typically focused on components of ICD systems, terminology, and capabilities.

They can implant a 3 lead with a defib as you stated...or deactive it and give you a S-ICD...defib only.

Generally appropriate

#### Programming

The quality of advice regarding the ICD programming, particularly pacing parameters, antitachycardia pacing, and arrhythmia detection algorithms, was mixed (7/16, 41%, appropriate; 3/17, 18%, inappropriate; and 6/17, 36%, without sufficient context).

After the MADIT-RIT study, ICDs are very rarely programmed to shock at heart rates lower than 200 or 220.

Generally appropriate

Comments coded as being without sufficient context included information regarding specific programming parameters and algorithms that may be appropriate in some, but not all, clinical circumstances.

AAIR (atrial rate adaptive) pacing may be preferable to DDDR (dual chamber rate adaptive) by avoiding an abnormal ventricular activation pattern.

The pacemaker part of your implant does not limit you to 80 bpm.

Without sufficient context

#### Cardiovascular Disease

The quality of information addressing cardiovascular disease was similarly mixed, with 56% (5/9) coded as generally appropriate, 33% (3/9) as without sufficient context to support, and 11% (1/9) as inappropriate.

SSS stands for sick sinus syndrome. The sinus node is the heart’s natural pacemaker. The SA node sends the electrical impulse to the atria to initiate a beat. When the SA node doesn’t work properly, the PM steps in.

Generally appropriate

Pieces of advice coded as being without sufficient context to support again pertained to information only accurate to some clinical situations.

You could be in the 10% to 13% of patients (depending on which scientific publication you read) whom experience early heart failure hospitalization associated with “conventional pacing.” Historic pacing bypasses the cardiac conduction system.

Without sufficient context

#### Lead Management

Only 4 instances including advice regarding lead management were identified, and these were split between being generally appropriate (2/4, 50%) and controversial (2/4, 50%). These comments were related to the advantages and disadvantages of lead extraction, a potentially high-risk procedure associated with ICDs.

They can be capped off and left there indefinitely. Extraction is a more specialized surgery, requiring an expert in the field and it has some risk. I would not do it unless it was necessary. There are no additional precautions to follow.

Generally appropriate

They do not have to leave leads in. I for one am not a damn junk yard and will not accept unused trash to be left behind...lead removal is quite common and not much of a big deal.

Controversial

#### Activity Restriction

Advice addressing whether or not patients with ICDs should avoid certain activities or environments was common (15 instances) and either generally appropriate (10/15, 67%) or generally inappropriate (5/15, 33%).

When people say 8 weeks, that’s for lifting heavy and raising the arm overhead. Most docs say 4-6 weeks for that. And other than those two limits—overhead and lifting heavy—you can and should use the arm normally

Generally appropriate

Instances in which commenters incorrectly advised patients to avoid small electrical devices (electric razors, tattoo needles, etc) were particularly common among those coded as generally inappropriate.

Just don’t get a tattoo directly over the device. Anywhere else is ok.

Generally inappropriate

#### Procedures

While less common (6 instances), advice related to procedures was more problematic. All instances in this category were determined to be either generally appropriate (2/6, 33%) or inappropriate for most patients (4/6, 67%). The specificity of the advice related to quality, with general advice being coded as appropriate and specific advice being inappropriate.

(In reference to a question regarding an upcoming noncardiovascular procedure) Just make sure the surgeon and anesthesiologist know in advance.

Generally appropriate

You were one of the less than 1% of PM patients that is inflicted with an infection. Should you need surgery again they will take extra precautions as a result. That makes the likelihood of another infection even less than 1% for you.

Generally inappropriate

#### Other Disease Management

Advice regarding other approaches to managing cardiovascular disease and arrhythmias varied considerably in terms of quality, with 23% (3/13) of such comments being coded as generally appropriate, generally inappropriate, or controversial and 31% (4/13) deemed to not have sufficient context to support. Within this category, more specific advice (eg, to begin or stop specific medications or vitamins) were likely to be categorized as generally inappropriate or controversial.

I would advise you to start taking some vitamins; I buy them from this web site that I found here: (redacted) and I buy from this site: (redacted) I don’t know if you can buy them from UK, but try to find similar ingredients. Also, doctors recommend to stay always hydrated which is mean to drink water with a bit sea salt or buy smart water that already have some ingredients.

Generally inappropriate

If your ICD was implanted because you were losing consciousness, removal of that device or turning it off could mean that you lose consciousness while driving and would possibly kill yourself and/or someone else. Also if your heart has actually stopped and an ICD was implanted to restart your heart, turning it off could have fatal consequences.

Controversial

## Discussion

This analysis of the quality of medical information exchanged between members of an ICD-specific Web-based message board provides unique insight into the quality and accuracy of the advice patients will find on such websites. An accurate understanding of the quality of this information is critical, as patients or caregivers will use Web-based resources to help navigate complex decisions regarding ICDs [4]. Because the use of Web-based resources is a common component of more general efforts to learn and guide disease self-management behaviors in cardiovascular care [4,5,7], providers can use these findings to help guide patients to appropriate, accurate, and helpful resources and warn them of dangers particular to others with inaccurate, decontextualized, or controversial advice.

While the quality of advice shared between members of an ICD-specific Web-based forum was mixed, half of such advice was generally appropriate. The proportion of appropriate advice differed among aspects of ICD treatment. As little as a quarter of the advice regarding other disease management and as much as two-thirds related to activity restriction was of generally good quality. In many cases, the quality of any individual piece of advice was inversely related to its specificity. That is, nonspecific and context-independent advice is of higher quality in this venue. Examples include descriptions of cardiovascular disease, the general utility of devices, and encouraging patients to discuss individual questions with their health care providers. Conversely, controversial or inappropriate advice featured prominently in more specific discussions, including those addressing specific device programming parameters (which vary depending on individual patient characteristics), and discussions of device and procedure risks. Interestingly, risks associated with device implant and lead extraction procedures tended to be understated, while risks associated with everyday activities (use of electronic devices in particular) were generally overstated.

In cases where members sought general information about ICDs and their functionality, the advice provided on this message board provides a succinct, accessible, and well-organized resource of basic information of interest to ICD patients and candidates. In this sense, anonymously submitted information appearing on this internet message board acts as a resource that might help avoid gaps in fundamental understanding among patients observed previously [16-17] and may provide a reliable reference to which providers can refer patients. Unfortunately, other recent investigations into how patients with cardiovascular disease act on the information they find on message boards suggest that the questions they seek answers to on Web are highly specific in nature [7], which in our sample were more likely to produce problematic information. In this way, health care providers may be best served to prospectively advise patients to avoid acting on Web-based information, which is highly context and patient specific.

While these findings are relevant to patient education, they should be considered within several limitations. First, we only analyzed conversations occurring on a single message board and the quality of information elsewhere may differ, including discussions occurring on social media platforms (eg, Facebook and LinkedIn) [5], which allow for conversations on member and organization pages in addition to dedicated message boards. The anonymity offered by the avatar-based system used on the site we analyzed may increase the honesty and frankness of discussion [18], but may alter the questions asked and advice provided if compared to a similar discussion occurring on Facebook. Second, we did not make any effort to determine whether any members had specific expertise that would influence the quality or accuracy of the advice they provide to other members. While no members identified themselves as health care providers during the project period, it may be possible that some members were providing information as they would to patients in a professional capacity. Nonetheless, these data may be representative of the quality of medical information appearing on many unmoderated, anonymously sourced message boards specific to cardiovascular and other treatment experiences.

## Abbreviations

ICD: implantable cardioverter-defibrillator
